# A Newly Discovered Phenylethanoid Glycoside from *Stevia rebaudiana* Bertoni Affects Insulin Secretion in Rat INS-1 Islet β Cells

**DOI:** 10.3390/molecules24224178

**Published:** 2019-11-18

**Authors:** Jing He, Nai-Liang Zhu, Jing Kong, Ping Peng, Lin-Fu Li, Xiao-Lu Wei, Yan-Yan Jiang, Yan-Ling Zhang, Bao-Lin Bian, Gai-Mei She, Ren-Bing Shi

**Affiliations:** 1 School of Chinese Materia Medica, Beijing University of Chinese Medicine, The Key Unit of Exploring Effective Substances of Classical and Famous Prescription of SATCM, Beijing 102488, China; hejing0932@163.com (J.H.); zhu13liang@126.com (N.-L.Z.); kj556776@163.com (J.K.); pengping2177@126.com (P.P.); lflfllf2001@126.com (L.-F.L.); jyyjm1129@163.com (Y.-Y.J.); collean_zhang@163.com (Y.-L.Z.); 2 Institute of Medicinal Plant Development, Chinese Academy of Medical Sciences, Beijing 200293, China; 3 Quality Standards, Key Laboratory of Beijing for Identification and Safety Evaluation of Chinese Medicine, Institute of Chinese Materia Medica, China Academy of Chinese Medical Sciences, Beijing 100700, China; xlwei@icmm.ac.cn (X.-L.W.); blbian@icmm.ac.cn (B.-L.B.)

**Keywords:** DS, insulin secretion, phenylethanoid glycoside, *Stevia rebaudiana* Bertoni, structure

## Abstract

The tea-like beverage *Stevia rebaudiana* Bertoni (*Stevia*) is popular in China because it reduces blood glucose and has a sweet taste. In this work, a comprehensive quality assessment of *Stevia* led to the discovery of five phenylethanoid glycosides, namely steviophethanoside (**1**), cuchiloside (**2**), salidroside (**3**), icariside D (**4**), and tyrosol (**5**). Of them, compound **1** is a novel compound. Mass spectrometry and NMR spectroscopy were employed to confirm the absolute configuration. A hydrolytic step with 4 N TFA at 95 °C for 4 h was used to confirm the monosaccharides. In addition, Discovery Studio 4.0 was used to predict the ADME and toxicity activity of compound **1**. The results suggested that compound **1** was biocompatible and had poor toxicity, which was verified by rat INS-1 islet β cells through an MTT assay. Meanwhile, a significant stimulatory effect on INS-1 cells was observed, which indicated a hypoglycemic effect of compound **1**. This is the first report that describes a natural, novel, and hypoglycemic phenylethanoid glycoside in *Stevia*.

## 1. Introduction

*Stevia rebaudiana* Bertoni (*Stevia*), has been used as a non-cariogenic sweetener for many years [1]. Early in the development of sweeteners, several non-cariogenic sweeteners, such as steviol glycosides, stevioside, and rebaudioside (A to F), were isolated from *Stevia*. Steviol glycosides have been used to replace sucrose in the treatment of diabetes mellitus and obesity [2]. Recently, several biological activities such as the beneficial effects of blood glucose regulation, antioxidant, and renal protective properties have attracted attention [3,4,5]. It is reported that the crude extract of *Stevia* had the ability to decrease blood glucose [6]. In addition, stevioside may indirectly contribute to anti-hyperglycemic action [7]. The aqueous extracts of the plant have been extensively used in Paraguay, and the clinical efficacy of regulating blood glucose was confirmed in related studies [8,9].

Normally, blood glucose was decreased by promoting insulin secretion, inhibiting glucose absorption, and promoting glucose decomposition. Among them, insulin is an important and effective tool to control hyperglycemia. *Stevia* can decrease plasma glucagon in diabetic rats by the mechanism of an improvement of insulin and suppression of glucagon level [7]. Recently, our group discovered that the polyphenolic compounds of *Stevia* could prevent diabetes and its associated complications in the streptozotocin rat and mouse model [10,11]. Compared with *Stevia* leaves, polyphenolic compounds decreased blood glucose more significantly [4]. What is more, a PK/PD-DI investigation displayed that some of the phenolic components may be absorbed into blood immediately. More interestingly, those compounds had the highest blood–drug concentrations at the drug effect time [11].

Hence, our previous research systematically studied the chemical constituents of the phenolic components in *Stevia* leaves [12]. In this process, a new type compound, phenylethanoid glycosides, was found in the phenolic components of *Stevia*. Phenylethanoid glycosides are widely distributed in the medicinal kingdom of plants. They are a type of phenolic compounds characterized by a phenethyl alcohol structure. Some of them are phenylethanoid and phenylpropanoid hybrids forming ester bonds with sugars [13]. Those structures contribute a broad range of biological activities and guarantee safety for phenylethanoid glycosides, which provides great potential in pharmaceutical applications. It is well known that phenylethanoid glycosides have strong antioxidant effects [14,15]. Phenylethanoid glycosides also can retard the occurrence of diabetic complications [16]. In recent years, many natural products with antioxidant effects have been shown to have hypoglycemic effects. More recently, the natural epicatechin was confirmed to modulate insulin secretion from pancreatic β cells [17,18]. Furthermore, Quercetin and oleic acid have a contribution to the insulin secretory dysfunction [19]. Quercetin-3-Oleoyl and berberine are potential novel agonists of fatty acid receptor GPR40 [20,21]. Natural products seem to become the preferred choice to deal with the chronic metabolic disorde. This raised our attention to find the connection between diabetes and phenylethanoid glycosides, which exist in the phenolic extract of *Stevia*.

Since *Stevia* can decrease plasma glucagon through the mechanism of an improvement of insulin, phenylethanoid glycosides are probably involved in promoting insulin secretion. Generally, the islet contains four different endocrine cell types, which play a major role in control of metabolic fuel homeostasis. Except the dysfunction of α-cells, δ-cells, and the pancreatic-polypeptide secreting cells (PP cells), a dysregulation of insulin secretion from β cells directly leads to type II diabetes [22]. INS-1 cell line is a widely used cell line for evaluating insulin secretion and β cell function in vitro. It was established in the culture medium, which contains 2-mercaptoethanol (2-ME). With the elevation of the glucose concentration, INS-1 cell line showed a steady increase in insulin secretion. Currently, there is no evidence that phenylethanoid glycosides promote insulin secretion. Therefore, exploring its hypoglycemic effect becomes interesting and important. This paper describes, in detail, the isolation, structural elucidation, and the stimulatory effect of phenylethanoid glycosides on insulin secretion.

## 2. Results and Discussion

### 2.1. Structure Elucidation

The dried leaves of *Stevia* were extracted twice with 50% EtOH. A new phenylethanoid glycoside (**1**) and four known compounds (**2**–**5**) were isolated and purified via AB-8 and Diaion HP-20 macroporous adsorptive resin, Sephadex LH-20 gel chromatograpy, and semi-preparative HPLC.

Steviophethanoside (**1**) was isolated as white amorphous powder, which showed the molecular ion peak in the high-resolution mass spectrum at *m*/*z* 455.1515, corresponding to the molecular formula C_19_H_28_O_11_ (calculated for C_19_H_28_O_11_Na: 455.1529). The ^1^H-NMR data at δ_H_ 6.82 (2H, d, *J* = 8.0 Hz, H-3, H-5) and 7.19 (2H, d, *J* = 8.0 Hz, H-2, H-6) showed a *p*-disubstituted benzene ring, as evidenced by the ^13^C-NMR data: δ_C_ 118.3 (d, C-3, C-5), 133.2 (d, C-2, C-6), 133.5 (s, C-1), 156.8 (s, C-4). In addition, the aliphatic proton signals at δ_H_ 2.89 (2H, t, *J* = 7.0 Hz, H-7), 3.84 (1H, dd, *J* = 12.0, 7.0 Hz, H-8a), 4.09 (1H, dd, *J* = 12.0, 7.0 Hz, H-8b), as well as ^13^C-NMR data δ_C_ 37.2 (t, C-7) and 74.0 (t, C-8) indicated one phenylethanoid skeleton. These spectral data were similar to Tyrosol [23].

Apart from the tyrosol moiety, compound **1** possessed two sugar units, as evidenced by two anomeric protons at δ_H_ 4.43 (1H, d, *J* = 8.0 Hz, H-1’) and 4.33 (1H, d, *J* = 7.6 Hz, H-1’’). The glucose signals at δ_C_ 105. 1 (C-1’), 75.9 (C-2’), 78.5 (C-3’), 72.2 (C-4’), 77.9 (C-5’), and 71.1 (C-6’), as well as the signal of C-8 being shifted downfield by 11.2 ppm to δ_C_ 74.0 (t) in **1**, were observed, which indicated the glucopyranosyl moiety being linked at C-8 in **1**. This deduction was confirmed by the 1H detected heteronuclear multiple bond correlation (HMBC) from the anomeric proton signal at δ_H_ 4.43 (1H, d, *J* = 8.0 Hz, H-1’) to δ_C_ 37.2 (t, C-7). The coupling value (*J* = 8.0 Hz) of anomeric proton suggested the presence of a β-glucopyranosyl moiety [24].

**Figure 1:**
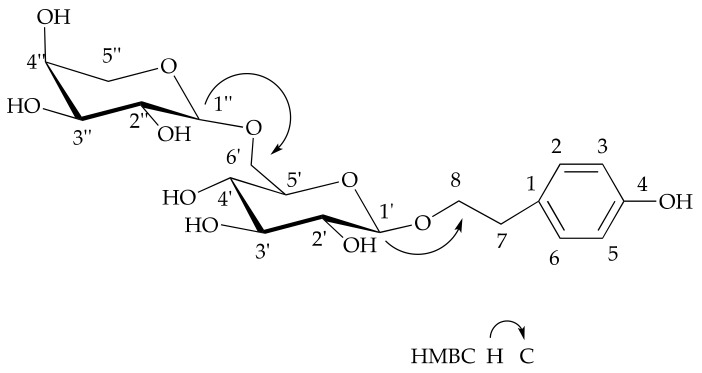
Structure and key HMBC correlations in 4-hydroxyphenyl ethyl-8-*O*-[α-l-arabinopyranosyl-(1→6)]-β-d-glucopyranoside.

Furthermore, the HMBC correlation observed from δ_H_ 3.78 (1H, dd, *J* = 11.7, 5.6 Hz, H-6’a) and δ_H_ 4.13 (1H, dd, *J* = 11.7, 5.6 Hz, H-6’b) to δ_C_ 106.5 (d, C-1’’) revealed that the other sugar unit was linked to C-6’. In addition, the ^13^C-NMR data δ_C_ 106.5 (C-1’’), 73.5 (C-2’’), 75.1 (C-3’’), 71.1 (C-4’’), and 69.1 (C-5’’) indicated that the other sugar unit was arabinopyranosy. The coupling constants (*J*_1’–2’_= 8.0 Hz) confirmed the moiety to be α-arabinopyranosyl, which suggests glycosylation at C-8 of aglycone with an ara (1→6)-glcmoiety. The absolute configuration of the monosaccharides was determined to be D-glu and L-ara through GC analysis of the chiral derivatives of the monosaccharides in the hydrolysate. The key HMBC correlations in **1** are shown in Figure 1. On the basis of all results, the structure of this new compound was established as 4-hydroxyphenyl ethyl-8-*O*-[α-l-arabinopyranosyl-(1→6)]-β-d-glucopyranoside.

Compound **2** was isolated as white amorphous powder. It is soluble in water and methanol, and has a positive response of FeCl_3_ and molish reagent. UV (MeOH − H_2_O) λ_max_ nm: 220.0, 279.1. ESI-MS^n^ *m*/*z*: 455 [M + Na]^+^, 431 [M − H]^−^, 455 (323), 431(299, 191, 179, 161, 149, 143, 131). ^1^H-NMR (500 MHz, D_2_O): δ_H_ 2.89 (2H, t, *J* = 6.8 Hz, H_2_-7), 3.25 (1H, t, *J* = 8.3 Hz, H-2’), 3.29 (1H, dd, *J* = 10.0, 6.4 Hz, H-2’’), 3.31 (1H, dd, *J* = 9.5, 3.0 Hz, H-5’’ a), 3.43 (1H, t, *J* = 8.5 Hz, H-4’), 3.46 (1H, t, *J* = 9.5 Hz, H-3’’), 3.47 (1H, t, *J* = 9.0 Hz, H-3’), 3.58 (1H, dd, *J* = 9.5, 6.5 Hz, H-5’), 3.63 (1H, ddd, *J* = 15.0, 9.8, 5.3Hz, H-4’’), 3.84 (1H, dd, *J* = 11.8, 5.8 Hz, H-6’ a), 3.89 (1H, dt, *J* = 9.5, 7.3 Hz, H-8a), 3.96 (1H, dd, *J* = 11.5, 5.5 Hz, H-5’’ b), 4.09 (1H, dt, *J* = 10.0, 7.0 Hz, H-8b), 4.14 (1H, d, *J* = 12.0 Hz, H-6’ b), 4.45 (1H, d, *J* = 9.0 Hz, H-1’’), 4.47 (1H, d, *J* = 8.5 Hz, H-1’), 6.88 (2H, d, *J* = 8.0 Hz, H-3, 5), 7.23 (2H, d, *J* = 8.0 Hz, H-2, 6). ^13^C-NMR (125 MHz, D_2_O): δ_C_ 37.2 (t C-7), 68.0 (t, C-5’’), 71.4 (t, C-6’), 72.1 (d, C-4’’), 72.3 (d, C-4’), 74.0 (t, C-8), 75.8 (d, C-2’), 75.9 (d, C-2’’), 77.8 (d, C-5’), 78.5 (d, C-3’’), 78.5 (d, C-3’), 105.1 (d, C-1’), 106.4 (d, C-1’’), 118.3 (d, C-3, 5), 133.2 (d, C-2, 6), 133.4 (s, C-1), 156.8 (s, C-4). The spectral data of compound **2** are consistent with the cuchiloside [25].

Compound **3** was isolated as white amorphous powder. It is soluble in water and methanol, and has a positive response of FeCl_3_ and molish reagent. UV (MeOH − H_2_O) λ_max_nm: 216.5, 273.2. ESI-MS^n^ *m*/*z*: 323 [M + Na]^+^, 323 (291, 237, 230, 209, 193, 121). ^1^H-NMR (500 MHz, D_2_O): δ_H_ 2.89 (2H, t, *J* = 5.7 Hz, H_2_-7), 3.25 (1H, t, *J* = 8.5 Hz, H-2’), 3.38 (1H, t, *J* = 9.3 Hz, H-4’), 3.42 (1H, d, *J* = 10.5 Hz, H-3’) 3.47 (1H, t, *J* = 9.0 Hz, H-5’), 3.72 (1H, dd, *J* = 12.3, 5.7 Hz, H-6’a), 3.88 (1H, d, *J* = 14.5 Hz, H-8a), 3.91 (1H, d, *J* = 12.0 Hz, H-8b), 4.09 (1H, t, *J* = 8.3 Hz, H-6’ b), 4.46 (1H, d, *J* = 8.0 Hz, H-1’), 6.87 (2H, d, *J* = 8.0 Hz, H-3, 5), 7.23 (2H, d, *J* = 7.5 Hz, H-2, 6). ^13^C-NMR (125 MHz, D_2_O): δ_C_ 37.2 (t, C-7), 63.6 (t, C-6’), 72.5 (d, C-4’), 73.9 (t, C-8), 76.0 (d, C-2’), 78.6 (d, C-5’), 78.8 (d, C-3’), 105.1 (d, C-1’), 118.3 (d, C-3, 5), 133.2 (d, C-2, 6), 133.4 (s, C-1), 156.8 (s, C-4). The spectral data of compound **3** are consistent with the salidroside [26].

Compound **4** was isolated as white amorphous powder. It is soluble in water and methanol. UV (MeOH − H_2_O) λ_max_nm: 218.9, 279.1. ESI-MS^n^ *m*/*z*: 323 [M + Na]^+^, 323 (302, 266, 248, 230, 205, 185, 180, 156, 145, 121). ^1^H-NMR (500 MHz, D_2_O): δ_H_ 2.86 (2H, t, *J* = 6.3 Hz, H_2_-7), 3.53 (1H, t, *J* = 9.3 Hz, H-4’), 3.58 (1H, d, *J* = 9.0 Hz, H-2’), 3.62 (1H, d, *J* = 7.5 Hz, H-3’), 3.65 (1H, d, *J* = 9.0 Hz, H-5’), 3.78 (2H, dd, *J* = 12.0, 5.5 Hz, H_2_-8), 3.84 (1H, t, *J* = 6.5 Hz, H-6’a), 3.96 (1H, d, *J* = 12.5 Hz, H-6’ b), 5.13 (1H, d, *J* = 7.5 Hz, H-1’), 7.13 (2H, d, *J* = 8.0 Hz, H-2, 6), 7.31 (2H, d, *J* = 8.0 Hz, H-3, 5). ^13^C-NMR (125 MHz, D_2_O): δ_C_ 39.8 (t, C-7), 63.4 (t, C-6’), 65.4 (t, C-8), 72.3 (d, C-4’), 75.8 (d, C-2’), 78.4 (d, C-5’), 78.9 (d, C-3’), 103.2 (d, C-1’), 119.5 (d, C-3, 5), 133.1 (d, C-2, 6), 136.7 (s, C-1), 157.9 (s, C-4). The spectral data of compound **4** are consistent with the icariside D [27].

**Figure 2:**
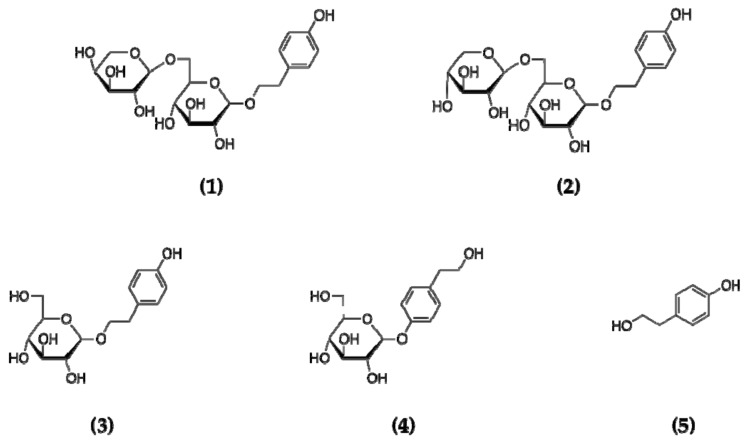
Structures of compounds steviophethanoside (**1**); cuchiloside (**2**); salidroside (**3**); icariside D (**4**); tyrosol (**5**).

Compound **5** was isolated as white amorphous powder. It is soluble in water and methanol. UV (MeOH − H_2_O) λ_max_nm: 221.2, 274.4. ESI-MS^n^ *m*/*z*: 161 [M + Na]^+^, 161 (141, 133, 121, 107, 91, 65). ^1^H-NMR (500 MHz, D_2_O): δ_H_ 2.79 (2H, t, *J* = 6.3 Hz, H-2), 3.79 (2H, t, *J* = 8.5 Hz, H-1), 6.87 (2H, d, *J* = 7.5 Hz, H-5, 7), 7.20 (2H, d, *J* = 8.0 Hz, H-4, 8). ^13^C-NMR (125 MHz, D_2_O): δ_C_ 36.9 (t, C-2), 62.8 (t, C-1), 115.4 (d, C-5, 7), 130.3 (d, C-4, 8), 131.0 (s, C-3), 153.8 (s, C-6). The spectral data of compound **5** are consistent with tyrosol [23] (Figure 2).

### 2.2. Pharmacokinetic Properties

The predicted pharmacokinetic properties include HIA (human intestinal absorption), BBB (blood brain barrier), PPB (plasma protein binding), CYP2D6, aqueous solubility, and toxicity. Our results showed that compound **1** was found to be non-hepatotoxic (Hepatotoxicity level of 0) and non-inhibitors of CYP2D6 (CYP2D6 level of 0). In addition, the binding was below 90% to carrier proteins. It is well known that the liver plays a critical role in transforming and clearing chemicals, and it is susceptible to the toxicity from these agents. Chemicals that cause liver injury may also induce the injury of other organs (like kidney). In early studies, traditional Chinese medicine presenting with high security and low toxicity compounds even without toxicity, such as phenylethanoid glycosides, which existed in purified fraction of goldengermander, were confirmed to show a lack of hepatotoxicity [28]. Combined with the current results of pharmacokinetic properties, compound **1** could be considered as the one that had low hepatotoxicity, good bioavailability [29], and involved less drug–drug interaction. In addition, the results revealed that compound **1** had optimal aqueous solubility of 4 with undefined BBB level at 4 and very poor absorption (HIA levels of 3), which might be considered as more hydrogen bond donors and acceptors. The property of high solubility in water was probably due to the lower log *p* value of the compounds, which depicted lower lipophilicity [30].

TOPKAT provides the capability to rapidly and confidently evaluate the toxic effects of chemicals directly from the molecular structure. In this study, compound **1** was found to be non-mutagenic (NM), non-carcinogenic (NC), and non-skin irritant in the toxicity parameter. Besides, the calculated LD_50_ (oral administration to rats) value of compound **1** was 5.61 g/kg. According to the different categories defined by the GHS, the category of compound **1** is higher than 5, which means that compound **1** is non-toxic after deglutition [31]. Although the prediction suggests that compound **1** may involve skin sensitization, skin, and ocular irritancy, i.e., some kind of developmental toxicity potential, the toxic dose is currently unclear (Table 1).

### 2.3. Cytotoxicity Assay

**Figure 3:**
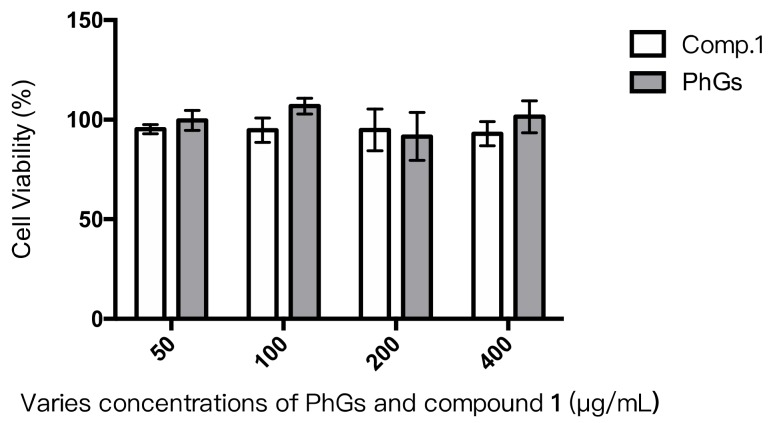
Cell viability of phenylethanoid glycosides (PhGs) and compound **1** on INS-1 cells.

To investigate the cytotoxic effects of compound **1**, an MTT assay was used. Regarding the similarity structure, phenylethanoid glycosides (PhGs) (including compound **1** and **2**) were evaluated together. After 48 h of exposure, no significant decreases in cell viability was observed dealing with various concentrations of compound **1** and PhGs (Figure 3). This means that both compound **1** and PhGs were not toxic to the INS-1 cells. The result indicated that compound **1** and compound **2** could not exert harmful effects on rat INS-1 cells even at the higher concentration of 400 μg/mL. Although there is no significant difference between compound **1** and PhGs, PhGs presented a trend of more secure effects than single compound **1**. Those results provided another proof of low toxicity.

### 2.4. Insulin Secretion in Vitro

**Figure 4:**
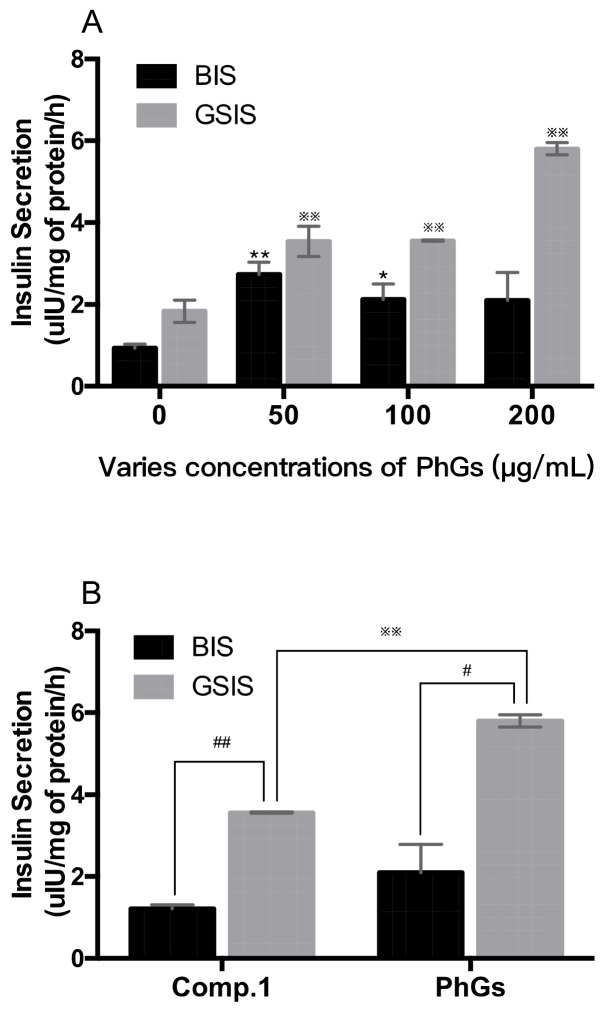
Effect of compound **1** and PhGs on glucose-stimulated insulin secretion from INS-1 cells. (**A**) Various concentrations of PhGs were described; the black columns show the BIS groups, the grey columns show the GSIS groups; (**B**) A 200 µg/mL dose between PhGs and compound **1** was portrayed. * *p* < 0.05, ** *p* < 0.01 compared with cells incubated with 2.8 mM glucose only (BIS groups); ^※※^ *p* < 0.01 compared with cells incubated with 16.7 mM glucose only (GSIS groups); ^#^ *p* < 0.05, ^##^ *p* < 0.01 compared with cells between BIS groups and GSIS groups.

Shivanna found that when rats were fed with whole leaves powder and extracted polyphenols of *Stevia*, a reduction of blood glucose, alanine transaminase (ALT), aspartate transaminase (AST), and increment of insulin level were observed [4]. In this study, the results for the glucose-mediated insulin release from INS-1 cells are presented in Figure 4. Substimulatory glucose (2.8 mM, BIS) showed less insulin release (0.99 µIU/mg of protein/h) than that (1.99 µIU/mg of protein/h) at high glucose levels (16.7 mM, GSIS).

Compound **1** and PhGs were investigated in this study. The results of PhGs group exhibited dose-dependent insulin release effects on INS-1 cells after treatment with the drug (50, 100, and 200 µg/mL) for 1 h, and the corresponding values were 2.74 ± 0.22, 2.12 ± 0.27, and 2.10 ± 0.49 µIU/mg of protein/h on 2.8 mM glucose, 3.54 ± 0.26, 3.56 ± 0.01, and 5.81 ± 0.11 µIU/mg of protein/h on 16.7 mM glucose, respectively (Figure 4A). The rate of basic insulin secretion (BIS) was only slightly changed relative to the rate found in the glucose stimulated insulin secretion (GSIS). In a high-glucose environment, there was a significant increase of insulin secretion in 200 μg/mL compared to the other two concentrations. Although both compound **1** and PhGs group stimulated insulin secretion of INS-1 cells, a significant difference in the rates of the two groups were observed at the same dose (200 µg/mL) of 16.7 mM glucose. These results are in agreement with those expected from hypoglycemic agents. The effects of phenylethanoid glycoside on insulin secretion have previously been reported with type 2 diabetic *db*/*db* mice [32]. The results demonstrated that the investigation could be a starting point for an interesting investigation about the potential antidiabetic activity of the new compound. In early studies, the hypoglycaemic chemicals of *Stevia rebaudiana* Bertoni have been reported to be steviol and polyphenols, known as secretagogues and facilitate insulin release, protecting pancrease, etc. [33,34,35]. This paper demostrated that phenylethanoid glycosides could directly stimulate insulin secretion dose dependently. Those discoveries are meaningful for the investigation of hypoglycemic active component group of *Stevia*.

## 3. Materials and Methods

### 3.1. Material and Chemicals

*Stevia* were collected from Ganzhou Julong High-tech Industrial Co., Ltd. in Jiangxi province, China, and were naturally dried at room temperature. Samples were identified by Assistant Prof. Chunsheng Liu, Department of Botany, Beijing University of Chinese Medicine. A voucher specimen (No. 20111107) has been deposited in the Herbarium of the Department of Phytochemistry, Beijing University of Chinese Medicine. Column chromatography was performed on Diaion HP-20 (Mitsubishi Heavy Industries, Ltd, Tokyo, Japan); Sephadex LH-20 column (Fuji Silysia Chemical Co., Ltd, Osaka, Japan); and Chromatorex ODS (Fuji Silysia Chemical Co., Ltd, Osaka, Japan), respectively. TLC was carried on silica gel G precoated plates (Qingdao Haiyang Chemical Co., Qingdao, China) with EtOAc-HOAc-H_2_O (3:1:0.1). The spots were detected by spraying with 10% H_2_SO_4_ ethanol solution followed by heating.

Rat INS-1 islet β cells were generously presented by Prof. BaoLin Bian (China Academy of Chinese Medical Science, Beijing, China). INS-1 cell culture medium RPMI 1640, 0.25% Trypsin-EDTA (Ethylene diamine tetraacetate), FBS, DMSO, penicillin, streptomycin, and supplements were purchased from Gibco (Grand island, NewYork, NY, USA). MTT was purchased from Sigma-Aldrich (St. Louis, MO, USA). RIA kit purchased from HuayingBio (Shenzhen, China). Glucose and β-mercaptoethanol were purchased from BEIJING DINGGUO CHANGSHENG BIOTECHNOLOGY CO.LTD (Beijing, China). RIPA Lysis buffer (Item No. c1053) and protease inhibitor (Item No. p1265) were purchased from Applygen technologies Inc. (Beijing, China). Culture plates, culture flasks, tubes, and cell scrapers (Item No. 3010) were purchased from Corning (One Riverfront Plaza Corning, NewYork, NY, USA).

### 3.2. Isolation and Purification of Compounds ***1***–***5***

The phenolic extracts of *Stevia* were enriched from the plant as described previously [36]. Compounds **1**–**5** were isolated from the phenolic extracts. In detail, the phenolic extracts were dispersed with H_2_O and then subjected to macroporous resin Diaion HP-20 eluted with a step gradient of H_2_O/MeOH (1:0→0:1) to obtain eight fractions (Fr. A–Fr. H). Fr. B (55.7 g) was separated by a Sephadex LH-20 column (5 × 80 cm), eluted with MeOH, and then purified with a preparative high-performance liquid chromatography, which was performed on a CXTH 3000 chromatograph (Beijing Chuangxintongheng Co., Ltd. Beijing, China) equipped with UVD detector. A YMC C_18_ column (250 × 20 mm, 5 μm) was used at a flow rate of 8 mL/min to give compound **2** (30 mg), **3** (25 mg), and **4** (20 mg). Fr. D (44.6 g) was separated via reverse-phase chromatography over C-18 silica gel, eluted with MeOH − H_2_O (100:0→70:30 100:0→50:50), and then purified through preparative HPLC elution using a MeOH/H_2_O/HCOOH (15:75:0.1 20:80:0.1 25:75:0.1) system. Finally, compound **1** (50 mg) and **5** (15 mg) were obtained.

### 3.3. Structure Validation and Property Prediction

#### 3.3.1. Identification of the Isolated Compounds

^1^H and ^13^C NMR spectra, as well as a DEPT spectra, were recorded in D_2_O with Bruker DRX-500 spectrometers operating at 500 MHz for ^1^H, and 125 MHz for ^13^C, respectively. Coupling constants were expressed in Hertz (Hz) and chemical shifts were given on a (ppm) scale with tetramethyl silane as internal standard. Positive ion ESI-MS and HR ESI-MS were recorded on an AutoSpec 3000 spectrometer (VG, Manchester, UK) with glycerol as the matrix. UV spectra were obtained on a UV 210A Shimadzu spectrometer. ESI-MS measurements were done by positive ionization with the capillary temperature set at 200 °C, Electrospray voltage 4.5 kV/cm; nitrogen was used as nebulizer gas at 20 arb. Collision-induced dissociation was optimized for each compound around 26–30%. The MS was recorded in the range of 80 to 1000 *m*/*z*.

#### 3.3.2. Hydrolysis of Phenylethanoid Glycoside

The phenylethanoid glycoside (5 mg) was heated in 4 N TFA (trifluoroacetic acid, aqueous solution, 3 mL) at 95 °C for 4 h. The reaction mixture was extracted with CHCl_3_ (3 × 3 mL). Each remaining aqueous layer was concentrated to dryness to yield a residue that was subsequently dissolved in pyridine (2 mL), and L-cysteine methyl ester hydrochloride (2 mg) was added to the solution. The mixture was heated at 60 °C for 2 h, chlorotrimethylsilane (0.3 mL) was added, and the mixture was further heated at 60 °C for 2 h. After the reactions had been performed, the supernatant was analyzed by GC, which was performed on an Agilent Technology 7890 (Agilent Technologies, Palo Alto, CA, USA). The conditions were as follows: The column temperature was maintained at 80 °C for 5 min, then increased from 80 to 280 °C at a rate of 25 °C/min, and finally maintained at 280 °C for 5 min; the carrier gas was N_2_ (1.4 mL/min); the split ratio was 1/20; the injection temperature was 250 °C; and the injection volume was 1 μL. The absolute configurations of the monosaccharides were confirmed to be all D-glu and L-ara by comparison of the retention times of the monosaccharide derivatives with those of the standard samples D-glu (13.489 min) and L-ara (17.376 min).

#### 3.3.3. ADMET Prediction

In this work, both ADMET (absorption, distribution, metabolism, excretion, and toxicity) and TOPKAT tests were performed using Discovery Studio^®^ 4.0 (Accelrys, San Diego, CA, USA) [37,38]. The Parameter values from six parts including aqueous solubility, human intestinal absorption (HIA), hepatotoxicity, blood brain barrier (BBB) penetration, cytochrome P450 2D6 inhibition, and plasma protein binding (PPB) were evaluated through ADMET descriptors algorithm [39]. In toxicity prediction, DS TOPKAT was used to evaluate toxicities under small molecules. Compound **1** was selected in models section containing screening for developmental toxicity potentials (DTP), rodent carcinogenicity, rat chronic oral lowest observed adverse effect level, rat oral LD 50, Ames mutagenicity, skin sensitization (GPMT), skin irritancy, and ocular irritancy. Ultimately, all the parameters were analyzed in order to predict the new compound’s properties.

### 3.4. Cell Culture and Cytotoxicity Assay

The INS-1 cells were grown in RPMI 1640 medium supplemented with 10% FBS, 50 μM β-mercaptoethanol, 100 U/mL penicillin, and 100 μg/mL streptomycin at 37 °C in a humidified atmosphere of 5% CO_2_. 

The potential cellular toxicity of compound **1** on INS-1 cells was assessed with the MTT method. It is based on the conversion of MTT into formazan crystals by living cells. In this paper, INS-1 (1 × 10^5^ cells per well) were cultured in a 96-well culture plate for 24 h at 37 °C in atmosphere of 5% CO_2_. Then, different concentrations of compound **1** were added for culture another 48 h. In parallel, different concentrations of phenylethanoid glycosides (PhGs), which contain compound **1** and compound **2**, were added. At the end of the treatment, supernatants were removed, a 5 mg/mL MTT was added, and incubated for an additional 4 h. The purple formazan crystals that developed from tetrazolium within the cells by the action of mitochondrial succinate dehydrogenase were extracted into DMSO. The absorbance was measured at 490 nm using a Multiskan Go microplate spectrophotometer (Thermo Fisher scientific, Waltham, MA, USA). All measurements were performed three times.

### 3.5. Insulin Secretion Assay

Insulin secretion by the INS-1 cells was studied with a slight modification of the method described before [40]. In brief, 2 × 10^5^ of INS-1 cells per well were grown in RPMI 1640 medium, which was supplemented with 10% (*v*/*v*) fetal calf serum, penicillin (100 U/mL), and streptomycin (0.1 mg/mL), at 37 °C with 5% CO_2_ in a humidified atmosphere. Prior to the experiment, the cells were washed three times with phosphate-buffered saline and then incubated in Krebs–Ringer bicarbonate HEPES buffer (KRBH), which contained 115 mM NaCl, 5 mM KCl, 1 mM MgCl_2_, 24 mM NaHCO_3_, 2.5mM CaCl_2_, and 10 mM HEPES. In order to measure insulin secretion, half-confluent cells in a 12-well cell plate were incubated for 2 h at 37 °C in the KRBH buffer. After that, different compounds with 2.8 mM (substimulatory concentration) or 16.7 mM (high concentration) glucose were added in the treatment groups for another 1 h; PhGs was added at various concentrations ranging from 100 to 400 μg/mL. Control experiments (with no compounds) were performed using KRBH buffer containing 2.8 mM and 16.7 mM glucose. Both medium and cell debris were separated, collected, and centrifuged at 4 °C, 700 g, 5 min. Total proteins per well were measured through cell debris to corrected insulin. Insulin secretion was assessed by radio-immunoassay (RIA) as the concentration of insulin in both the culture medium and the cell debris.

### 3.6. Statistical Analysis

Values are given as the mean ± SEM, and n values refer to the number of independent experiments. In the case of representative experiments, values are the mean ± SD. The significance of differences was assessed by t test for unpaired data.

## 4. Conclusions

A new phenylethanoid glycoside, 4-hydroxyphenyl ethyl-8-*O*-[α- l-arabinopyranosyl-(1→6)]-β-d-glucopyranoside, and four phenylethanolyl glycosides were isolated from the leaves of *Stevia*. The new compound **1** showed moderate properties for its low toxicity on both DS prediction and cytotoxicity assay. In addition, a significant stimulatory effect was observed on the INS-1 cell line. In summary, this is the first report that describes a natural, novel phenylethanoid glycoside in *Stevia*. The research is a starting point for further investigation into the potential anti-diabetic activity of steviophethanoside, as well as the investigation of hypoglycemic active component group of *Stevia*.

## 5. Patents

Ren-bing Shi has a pending patent application “Preparation, activity, application and quality control of a new compound”, CN 104447900 A.

## Figures and Tables

**Table 1:** Absorption, distribution, metabolism, excretion, and toxicity (ADMET) and TOPKAT prediction of steviophethanoside.

Predicted Item	Compound 1
Absorption level (2–4)	3
BBB level (2–4)	4
Solubility level	4
CYP2D6 prediction (False-non inhibitor)	FALSE
Hepatotoxic prediction (False-non toxic)	FALSE
PPB Prediction (False-poorly bound)	FALSE
Mutagen	Non-Mutagen
Rodent Carcinogenicity	Non-Carcinogen
Skin irritancy	Non-Irritant
Skin sensitization	0.765
Rat oral LD_50_ (g/kg)	5.61
Ocular Irritancy	0.84
DTP (Developmental Toxicity Potential)	0.861
